# Unraveling a Case of Uncommon Shoulder Septic Arthritis Attributed to Burkholderia pseudomallei: Investigating a Mysterious Organism

**DOI:** 10.7759/cureus.69105

**Published:** 2024-09-10

**Authors:** Tathagath Tiwary, Vijayanand B, Rishab C

**Affiliations:** 1 Department of Orthopedics, Sri Ramaswamy Memorial (SRM) Medical College Hospital and Research Centre, Chennai, IND; 2 Department of Orthopedic Surgery, Sri Ramaswamy Memorial (SRM) Medical College Hospital and Research Centre, Chennai, IND

**Keywords:** advanced diagnostic technologies, diabetes mellitus, infectious diseases, melioidosis, rare case report, septic arthritis

## Abstract

Melioidosis is a serious infection caused by *Burkholderia pseudomallei*, typically found in tropical regions but now being increasingly recognized in areas outside its traditional endemic zones. This case report details the experience of a patient with type 2 diabetes mellitus who presented with unusual symptoms, complicating the diagnostic process. Initial treatment attempts were unsuccessful; however, the use of advanced microbiological methods allowed for the swift identification of the pathogen and led to effective treatment. The report showcases the critical need to include melioidosis in the differential diagnosis of severe infections, especially in patients with preexisting medical conditions or those in and around the endemic areas. It highlights the importance of timely and precise diagnosis, targeted antimicrobial therapy, and continuous monitoring to enhance patient outcomes.

## Introduction

*Burkholderia pseudomallei* is the pathogen responsible for melioidosis, an infectious disease predominantly found in tropical regions of Southeast Asia and Northern Australia [1]. The majority of the cases have been reported in Thailand and Singapore. The broad spectrum of symptoms associated with melioidosis, which can resemble those of other infections or conditions, complicates the diagnostic process [2]. In areas where melioidosis is endemic, there is a notable association with diabetes mellitus, which significantly heightens the risk of severe infections [3].

*B. pseudomallei* is a gram-negative, nonfermenting rod found in environmental sources such as soil and water [4]. It is primarily transmitted through contaminated water. Accurate global incidence rates of melioidosis are challenging to determine due to substantial underreporting. However, a spatial modeling study published in early 2016 estimated approximately 165,000 cases of melioidosis annually, with about 89,000 fatalities, indicating a mortality rate exceeding 50% [5]. This serious illness can present as acute, subacute, or chronic. Pneumonia is observed in around half of the patients at the time of admission. Other possible manifestations include genitourinary infections, skin infections, primary bacteremia, musculoskeletal infections such as septic arthritis and osteomyelitis, and central nervous system involvement [6,7].

This case report details the experience of a 55-year-old man with type 2 diabetes mellitus who developed a severe and rapidly worsening shoulder infection. Despite initial treatments, the patient’s condition deteriorated until *B. pseudomallei* was identified as the source of the infection. This case underscores the necessity of considering uncommon pathogens in diagnosing severe infections, particularly in diabetic patients who may exhibit atypical symptoms. Through this case, we aim to highlight the global significance of melioidosis and stress the importance of employing advanced diagnostic methods and targeted treatment to enhance patient outcomes.

## Case presentation

A 55-year-old male farmer with a history of type 2 diabetes mellitus presented with progressive right shoulder pain of one month's duration, described as insidious, rapidly intensifying, throbbing, and burning. The pain was nonradiating and worsened with shoulder movements. One week before admission, the patient developed a low-grade, continuous fever and jaundice, which had been managed at an outside facility.

On examination, the patient appeared thin, febrile, dehydrated, hypotensive, and pale. The right shoulder exhibited diffuse swelling, redness in the anterior aspect, warmth, tenderness, and painful movements. Initial resuscitation involved intravenous fluids to address dehydration and hypotension, antipyretic to manage fever, and insulin for elevated capillary blood glucose levels. Specific interventions included fluid boluses and vasopressor support to stabilize blood pressure.

**Figure 1:**
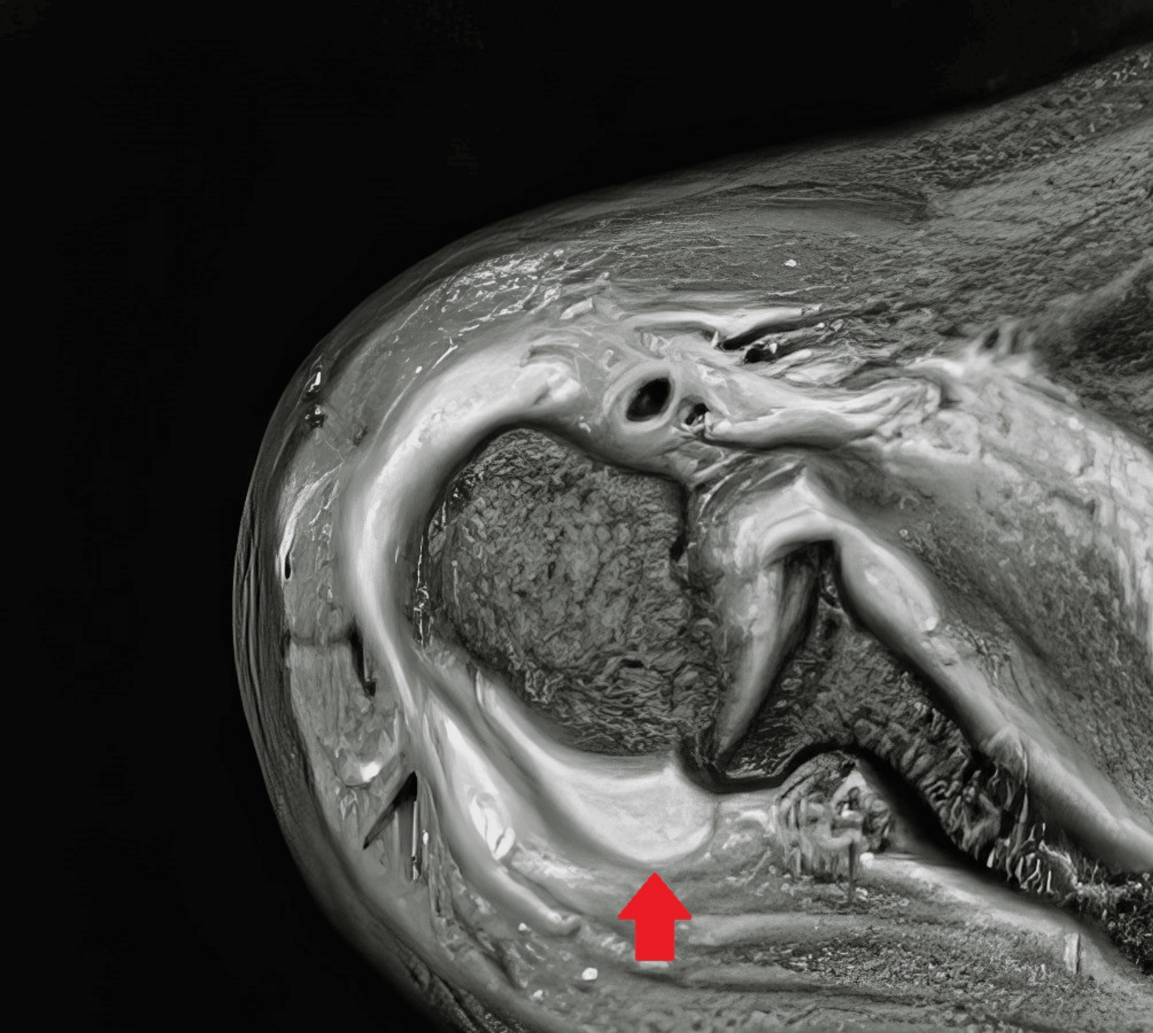
MRI of the right shoulder showing collection in the joint (red arrow) MRI: magnetic resonance imaging

Pus was collected through a stab incision and sent for culture. Routine blood investigations showed anemia (hemoglobin 4.9 g/dL), increased white blood cell count (19,400), and significantly raised C-reactive protein (>192 mg/L). His random blood sugars were 233 mg/dL, and hemoglobin A1C was 9.1%. Sodium levels were low at 127 mEq/L, and urine ketones were positive. Magnetic resonance imaging of the right shoulder (Figure 1) revealed pus collection in the joint space and surroundings. Additional imaging, such as radiograph, was performed. The patient was taken to the intensive care unit for further management.

Initial empirical treatment with intravenous piperacillin/tazobactam was initiated based on the broad-spectrum coverage of common pathogens. The initial culture showed Pseudomonas species. The antibiotics were continued. However, the patient’s condition deteriorated.

**Figure 2:**
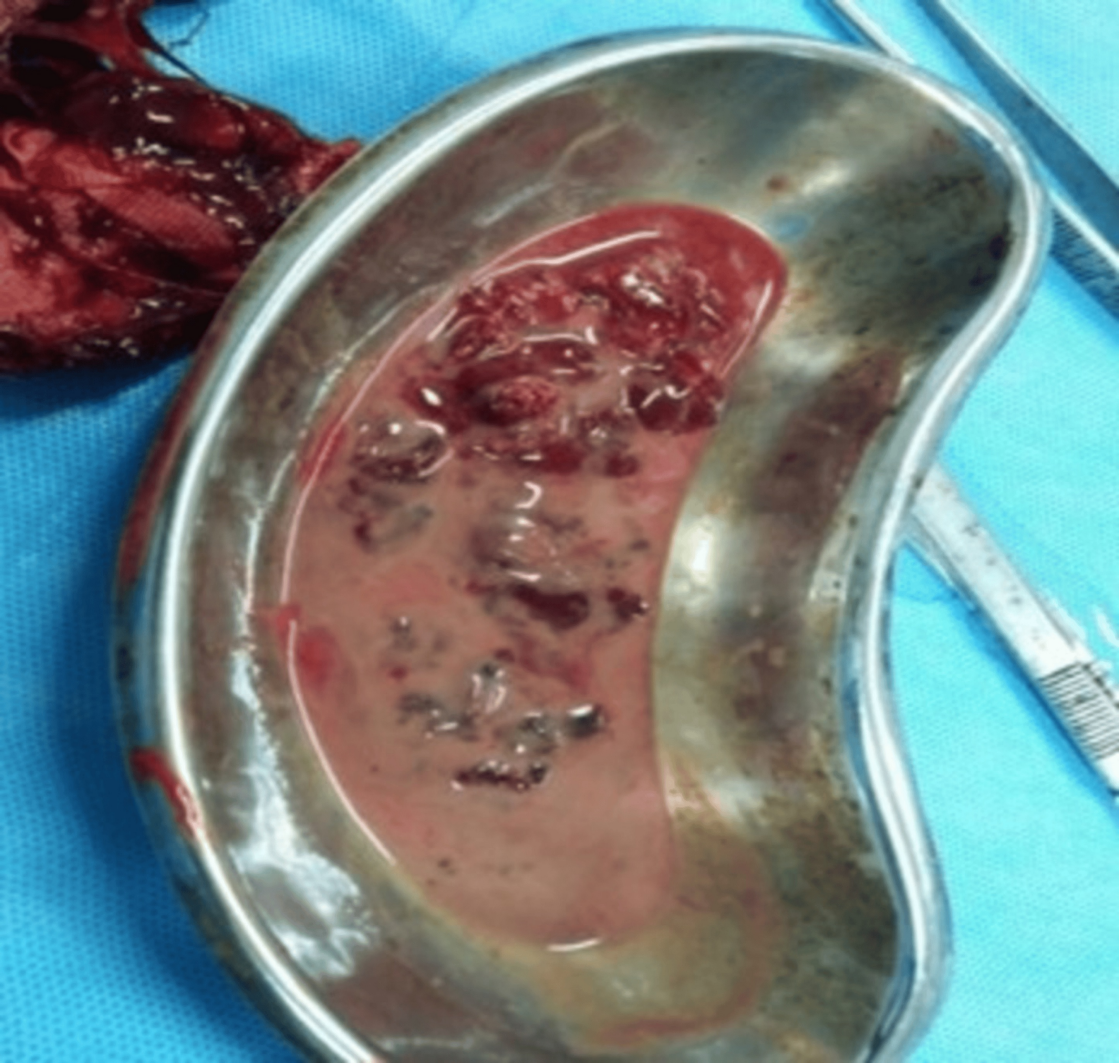
Pus obtained from the joint space

The patient underwent an emergency arthrotomy, and collections from the surgery (Figure 2) were sent for cultures along with blood cultures. The repeat cultures revealed gram-negative nonfermenting rods. However, this report was nonspecific as gram-negative nonfermenting is a broad term. On the recommendation of the microbiologist, the Vitek-2 system (bioMerieux, Salt Lake City, UT), an automated microbiological diagnostic tool, was employed to identify the specific pathogen on the recommendation of the microbiologist.

Vitek-2 revealed *B. pseudomallei* as the organism in pus and blood samples. The choice of piperacillin/tazobactam was reconsidered due to the lack of efficacy against *B. pseudomallei*, leading to a switch to intravenous ceftazidime 1 g, which is effective against this pathogen. The patient's condition improved with normalization of blood glucose and sodium levels. Ceftazidime therapy was continued for two weeks. Postoperatively, the patient experienced wound gaping on the fifth day, which was managed with regular dressing changes. The wound eventually healed well.

## Discussion

This case highlights a notable instance of *B. pseudomallei* infection in a patient with type 2 diabetes mellitus, aligning with existing literature on the challenges of diagnosing melioidosis. *B. pseudomallei*, an environmental bacterium, is typically associated with tropical regions and is acquired through inhalation, ingestion, or cutaneous inoculation [8].

*B. pseudomallei* infection is notably more common in males, which aligns with this case [9-11]. The patient's occupation as a farmer increases the risk of exposure to *B. pseudomallei*, commonly found in soil and water in endemic regions. This underscores the importance of considering occupational factors when evaluating patients with symptoms suggestive of melioidosis [12-14].

Type 2 diabetes mellitus significantly heightens the risk of severe melioidosis, as chronic hyperglycemia compromises immune function, thereby increasing vulnerability to serious infections [6,15-21]. The patient's severe shoulder pain with systemic symptoms is consistent with studies indicating that melioidosis can present as an atypical infection, particularly in immunocompromised individuals [18].

Utilizing the Vitek-2 system for pathogen identification was crucial in selecting the appropriate treatment for the patient, as it provided rapid and accurate identification of the pathogen, enabling a switch to the appropriate antimicrobial therapy. The Vitek-2 system employs a combination of biochemical tests to identify bacterial species. It operates by inoculating a microbial suspension into cards containing a variety of substrates that test for specific metabolic activities of the bacteria. The system then measures changes in color or turbidity, which indicate the presence or absence of these metabolic activities. These data are analyzed by the system's software, which compares it to a database of known bacterial profiles to provide accurate species identification [22]. Transitioning from piperacillin/tazobactam to ceftazidime was essential, as ceftazidime is recommended for treating melioidosis [23,24]. The patient’s rapid deterioration despite initial treatment highlights the aggressive nature of *B. pseudomallei* infections and the challenges in diagnosing rare infections.

The differential diagnosis for severe shoulder infections includes common pathogens such as *Staphylococcus aureus* and Streptococcus species [18]. However, the unusual progression and systemic symptoms in this case necessitated further investigation. Melioidosis can be easily misdiagnosed due to its broad range of clinical presentations [24]. Surgical intervention was necessary to address the source of infection directly and prevent further systemic involvement.

## Conclusions

The significance of taking *B. pseudomallei* into account when making a differential diagnosis of severe illnesses is highlighted in this case report. Melioidosis, although predominantly associated with tropical regions, poses a significant diagnostic challenge and requires a high index of suspicion, especially when conventional treatments fail to yield expected outcomes. Promptly identifying the pathogen through advanced microbiological techniques and the transition to targeted therapy played a crucial role in the patient's recovery. This case highlights the need for healthcare providers to remain vigilant for rare and potentially life-threatening pathogens, even in nonendemic areas.
